# High-density surface EMG maps from upper-arm and forearm muscles

**DOI:** 10.1186/1743-0003-9-85

**Published:** 2012-12-10

**Authors:** Monica Rojas-Martínez, Miguel A Mañanas, Joan F Alonso

**Affiliations:** 1 Biomedical Research Networking Center in Bioengineering, Biomaterials and Nanomedicine (CIBER-BBN), Barcelona, Spain; 2 Biomedical Engineering Research Centre (CREB), Barcelona, Spain; 3 Barcelona College of Industrial Engineering (EUETIB), Barcelona, Spain; 4 Department of Automatic Control (ESAII) Universitat Politecnica Catalunya, Barcelona, Spain

**Keywords:** High-Density surface electromyography, 2D electrode arrays, EMG pattern recognition, Artifact detection, Rehabilitation, Robotics, Prosthetics

## Abstract

**Background:**

sEMG signal has been widely used in different applications in kinesiology and rehabilitation as well as in the control of human-machine interfaces. In general, the signals are recorded with bipolar electrodes located in different muscles. However, such configuration may disregard some aspects of the spatial distribution of the potentials like location of innervation zones and the manifestation of inhomogineties in the control of the muscular fibers. On the other hand, the spatial distribution of motor unit action potentials has recently been assessed with activation maps obtained from High Density EMG signals (HD-EMG), these lasts recorded with arrays of closely spaced electrodes. The main objective of this work is to analyze patterns in the activation maps, associating them with four movement directions at the elbow joint and with different strengths of those tasks. Although the activation pattern can be assessed with bipolar electrodes, HD-EMG maps could enable the extraction of features that depend on the spatial distribution of the potentials and on the load-sharing between muscles, in order to have a better differentiation between tasks and effort levels.

**Methods:**

An experimental protocol consisting of isometric contractions at three levels of effort during flexion, extension, supination and pronation at the elbow joint was designed and HD-EMG signals were recorded with 2D electrode arrays on different upper-limb muscles. Techniques for the identification and interpolation of artifacts are explained, as well as a method for the segmentation of the activation areas. In addition, variables related to the intensity and spatial distribution of the maps were obtained, as well as variables associated to signal power of traditional single bipolar recordings. Finally, statistical tests were applied in order to assess differences between information extracted from single bipolar signals or from HD-EMG maps and to analyze differences due to type of task and effort level.

**Results:**

Significant differences were observed between EMG signal power obtained from single bipolar configuration and HD-EMG and better results regarding the identification of tasks and effort levels were obtained with the latter. Additionally, average maps for a population of 12 subjects were obtained and differences in the co-activation pattern of muscles were found not only from variables related to the intensity of the maps but also to their spatial distribution.

**Conclusions:**

Intensity and spatial distribution of HD-EMG maps could be useful in applications where the identification of movement intention and its strength is needed, for example in robotic-aided therapies or for devices like powered- prostheses or orthoses. Finally, additional data transformations or other features are necessary in order to improve the performance of tasks identification.

## Background

The electromyographic (sEMG) signals detected on the skin above human muscles are important to infer motion intention and therefore could be used to control devices such as exoskeletons, biofeedback systems or assistive tools [1,2]. Central to these goals is the extraction of information from myoelectric signal which is commonly detected with electrode pairs. Other purposes include the estimation of muscle force, biofeedback of the activity of the muscle and the analysis of myoelectric fatigue.

The main disadvantage of bipolar signals is that its amplitude depends on the distance between the active motor units and the recording electrodes [3]. Due to the low spatial resolution of the bipolar signal, the standard surface EMG reflects the activity of a number of motor units (MU) within a delimited area of the muscle. However, amplitude variations are expected in both, the parallel and the perpendicular directions of propagation of the MU action potentials (MUAP): in the former, the amplitude of the signal varies with the proximity to innervation zones and tendons, while in the latter the amplitude is attenuated because of the propagation properties of the conductor volume [4]. As pointed out by Zwarts et al. in [5], single channel approaches do not reflect the physical propagation of the potentials and therefore only the time-varying properties of the signals are usually analyzed, disregarding important spatial aspects of the propagation like extent and length of the muscle fibers, which are essential for the force-generating capacity of the muscle, and, if not well addressed can lead to incorrect conclusions. In recent years, on the other hand, the development and application of electrode arrays in one or two dimensions have allowed the study of the sEMG signal in the temporal and the spatial domain, opening new possibilities to the study of the neuromuscular system [5-7] and to the field of myoelectric control [8].

What is more, recent studies have demonstrated that the muscles do not activate homogeneously, that is, distinct regions of the muscle are activated differentially depending on the position of the joint [9] and the duration [10] and strength of the contraction [11]. Such activation may be related to bundles of fiber types organized in different regions within the muscle, each of them following different recruitment strategies according to Henneman’s size principle [11].

Therefore, EMG amplitude information provided by a single bipolar channel is highly dependent on the location of the recording electrodes, even when they are well located away from innervation zones and tendons and it does not offer the possibility of tracking inhomogeneities in the activation of the muscles.

The recording of sEMG signals with 2D arrays in a wide area of the muscles and the processing of the signal in the space dimension [5,12] can overcome some of the drawbacks of single-channel approaches, providing a quantification of the temporal and spatial properties of the electrical muscle activity [13,14]. In this study, High Density surface EMG (HD-EMG) signals recorded with 2-dimensional (2D) arrays of closely spaced electrodes were used to calculate activation maps for the upper arm and forearm. These maps provided a larger amount of information related to the tracking of (task changing) skin surface areas where EMG amplitude is maximal and a better estimation of muscle activity by the proper selection of the most significant channels. The main objective is to analyze patterns in the activation maps associating them with four movement directions at the elbow joint and with different strengths of those movements. Although the activation pattern can be assessed with bipolar electrodes, HD-EMG signals from upper-arm and forearm muscles could enable the extraction of features that depend on the spatial distribution of the MUAPs and on the load-sharing between muscles in order to have a better differentiation between tasks and effort levels.

For this purpose, two additional automatic algorithms are proposed, one intended for the automatic detection of low-quality signals and the other, for the segmentation of global activation areas. In the first case, different algorithms based on the spatial spread of the potentials over the skin surface have been proposed in the literature [9,15,16]. Such algorithms considered only the information of the bulk of data for the detection of outliers in 2D multichannel recordings. The approach proposed here takes into account features extracted from channels in the close- proximity neighborhood to evaluate the quality of a given signal and to interpolate its value from neighboring channels when needed. Besides, the method was designed to compromise between precision and sensitivity of the detection in order to reduce the number of misclassifications of good-quality signals.

In the second case, watershed segmentation has been previously proposed for determining the regions of activity in HD-EMG recordings [9], however this method is prone to over-segmentation in the presence of multiple discontinuities [17], particularly, those introduced by dissimilarities in the electrode-skin impedance of the different channels. The segmentation proposed here offers an alternative, being less sensitive to local maxima. Results show that features extracted from HD-EMG maps could be useful in the identification of movement intention.

## Methodology

### Instrumentation

Three 2-D electrode arrays were fabricated in hydrophobic fabric for the recording of high density surface EMG signals (HD-EMG). The arrays were developed in our laboratory and consisted in silver-plated and gel-filled eyelets (external diameter of 5 mm) equally spaced by 10 mm in rows (*y* in the proximal-distal direction) and columns (*x* in the medial-lateral direction). Textile fabric adapts to the geometry of the muscle while maintaining inter-electrode distance and good adhesion to the skin provided by elastic straps. Array 1 (forearm) had 6 rows and a variable number of columns (between 17 and 19) depending on the dimensions of the limb of the subject whereas arrays 2 and 3 (upper arm) consisted of 120 electrodes distributed in 8 rows × 15 columns. About 350 channels were recorded for each subject and were sufficient to cover the muscles of interest.

**Figure 1:**
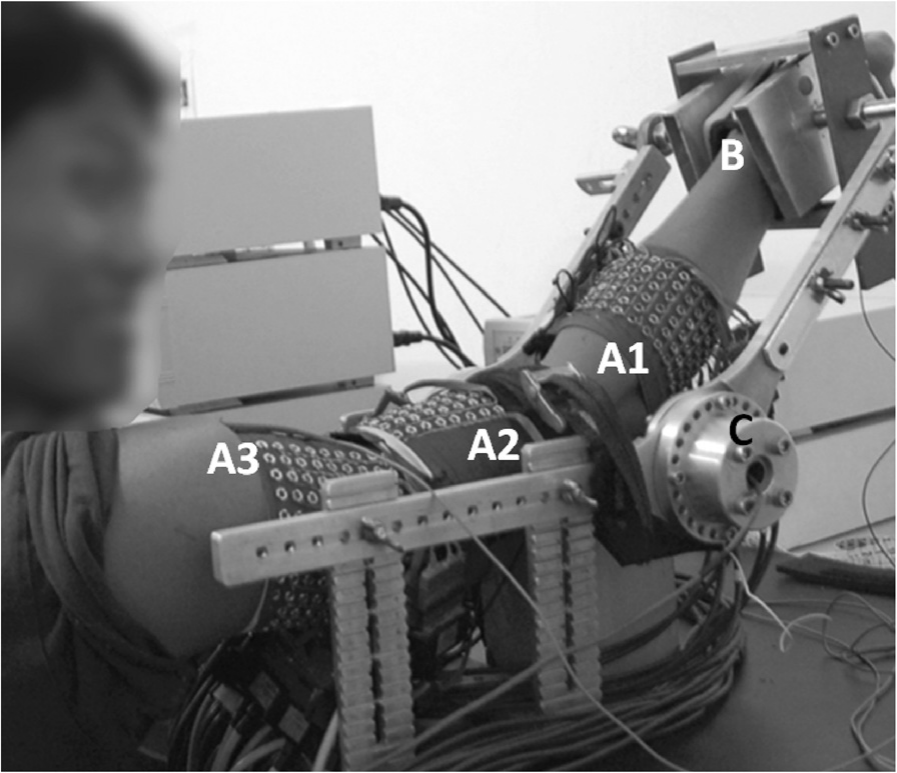
**Experimental set-up. **The three arrays (**A1**, **A2** and **A3**) and the mechanical brace can be observed. The forearm is held distally to avoid the action of the muscles of the wrist and hand (**B**) The exerted force is measured by transducers located at the joint of the mechanical brace (**C**).

High Density monopolar signals were recorded simultaneously by three synchronized amplifiers (EMG-USB- 128 channels, sampling frequency of 2048 Hz, 3dB bandwidth 10–750 Hz, programmable gains of 100, 200, 500, 1000, 2000, 5000 and 10000, LISiN-OT Bioelettronica). Power line interference was reduced by using a “driven right leg” (DRL) circuit [18] and reference electrodes were placed at the clavicle, wrist, and shoulder of the same (dominant) side. The force exerted in the four directions of movement (flexion, extension, pronation and supination of the forearm) was measured by two torque transducers (OT Bioelettronica, range 150 N.m, resolution 2.5 mV/V) located at the joints of a mechanical brace and aligned with the joint rotational axis. This brace also included an adjustable wrist lock at the end of the bars, in order to avoid hand gripping and wrist flexion/extension efforts (Figure 1). Torque signals were displayed on real time for visual feedback of the exerted force.

### Experimental protocol

Twelve healthy male volunteers (age, 28.3 ± 5.5 years; height: 177.8 ± 6.0 cm; body mass: 75.7± 8.7 kg) participated in the experiment. Subjects did not have any history of neuromuscular disorders, pain or regular training of the upper limb. All subjects gave informed consent to participate to the experimental procedure.

Five muscles associated with flexion, extension, pronation and supination of the forearm were selected for the study: Biceps and Triceps Brachii in the upper arm and Anconeus, Brachioradialis and Pronator Teres in the forearm. Array 1 (A1) was located on the forearm, with the most proximal electrode row 2 cm below the elbow crease, and was intended for covering the three forearm muscles whose edges were previously drawn over the surface of the skin according to Kendall et al. [19]. The columns of the array laid along the axis oriented from lateral to medial direction in order to cover each selected muscle with at least three columns. Array 2 and array 3 (A2 and A3) were located in the distal and proximal regions of the upper arm, respectively, with their centers on the location recommended by SENIAM [20]. Previous to the positioning of the electrode array, the skin was shaved and cleaned with abrasive paste. Conductive gel (20 μl) was inserted in each electrode of the array using a gel dispenser (Multipette Plus, Eppendorf, Germany). Lengths and circumferences of the upper arm and forearm segments were measured for each subject. The length of the ventral side of the upper-arm was measured from the acromion to the fossa cubit whereas the length of its dorsal side was measured from the posterior crista of the acromion to the olecranon. The length of the forearm was measured from the medial epicondyle to the apophysis of the radius. Circumferences of the arm segments were measured while contracting different muscles: the distal and proximal upper arm circumference were measured over the muscle belly of biceps and triceps respectively and the proximal forearm circumference was measured over the muscle belly of the Brachio Radialis (approximately 2 cm below the elbow crease). During the experiment, subjects sat in front of the mechanical brace with the back straight, the elbow joint flexed at 45°, shoulder abducted at 90° (arm in the sagittal plane), and forearm rotated 90°, midway between supination and pronation (Figure 1). Subject’s Maximal Voluntary Contraction (MVC) during flexion, extension, pronation and supination were obtained at the beginning of the experiment as the maximum of three trials for each task. Afterwards, subjects carried out a series of isometric contractions at 10%, 30% and 50% MVC. Contractions were performed in randomized order, each lasting for 10 seconds followed by 2 minutes rest in order to avoid cumulative fatigue. In addition, subjects were previously trained to maintain the hand and fingers at rest during signal recording.

### Detection of low quality signals

#### Features extraction

When recording a large number of physiological signals it is very likely to observe some low quality channels affected by artifacts originated in deformations of the skin under the electrodes, movement of the cables or in bad contacts between each recording electrode and the skin, inducing capacitive couplings and enabling power line interference [21]. Visual inspection of the outliers (channels with low-quality signals) is time-consuming and depends on the expertise of the operators. Thus, it becomes necessary to apply an automatic method to identify such signals (and perform adequate processing if necessary) before any kind of information extraction.

**Figure 2:**
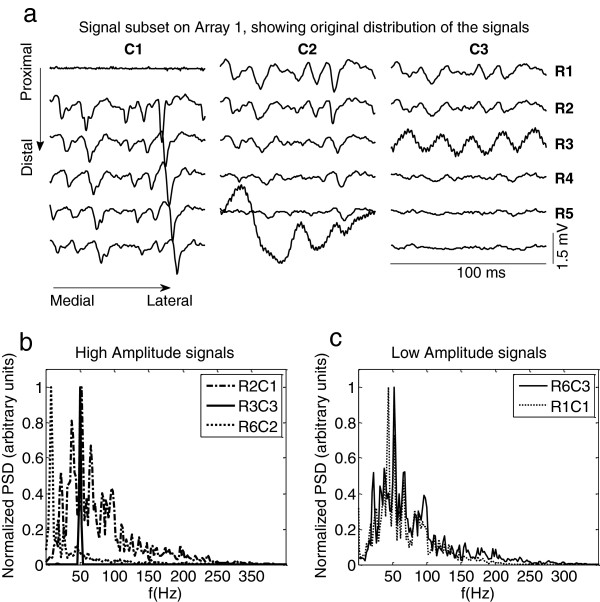
**a) Signal subset recorded in array A1 (forearm) during elbow flexion at 50% MVC. **Three columns (**C1** to **C3**) and six rows (**R1** to **R6**) are shown. Different kinds of artifacts are observed in C1R1, C2R6, and C3R3. It is also possible to observe that the energy of the signals changes in both x and y directions. Normalized Power Spectral Density for different signals are displayed at the bottom. Each spectrum was normalized with respect to its peak value. Artifactual channels can present higher or lower amplitude when compared to neighbor channels. **b**) High amplitude signals. It is possible to observe power-line components on the artifactual channel R3C3 and low frequency components on the artifactual channel R6C2. The attenuation of the analogue filter in the amplifier is not sufficient to remove the movement artifact on channel R6C2. **c**) Low amplitude signals. Thought normalized PSD is similar for R6C3 and R1C1, the later can be identified as an artifact when compared to neighbor channels in the temporal domain.

An example of low-quality signals is presented in Figure 2. They are characterized by: a) high-power low frequency components (R6C2, Figure 2b), b) high power components at power-line harmonics due to high-impedance contacts and stray capacitive couplings (R3C3, Figure 2b), and c) their energy may be much higher or lower than that of neighboring monopolar channels (R1C1, Figure 2c), especially if parallel-fibber muscles are considered. Figure 2c constitutes an especial case where the normalized power spectrum of the signals in R1C1 is similar to that corresponding to a non-artifact; however its amplitude is much lower when compared to its neighbors. Signals R4, R5 and R6 in column C3 have amplitude similar to R1C1, but they are probably located over a region of the limb with lower activation and cannot be considered as artifacts. Therefore, a detection system should take into account spectral and amplitude features and refer them not only to the bulk of data but also to its neighborhood.

For this purpose, three features for the detection of artifactual signals were defined:

1. Relative power of low frequency components *P*_*l/t*_, from 0 to 12 Hz

2. Relative power of power-line components *P*_*line/t*_ corresponding to 50Hz and its first four harmonics, and

3. Signal power calculated from the root mean square (RMS) of the signal

The Power Spectral Density of the signal was estimated with the FFT in non-overlapping signal epochs of 500 ms. Features (*P*_*l/t*_, *P*_*line/t*_ and *RMS)* were computed for each channel as the mean of the values obtained from six epochs over segments of 3s where the exerted force remained constant.

#### Automatic algorithm for artifact removal

An expert system based on thresholds associated with the three features described previously was designed.

**Figure 3:**
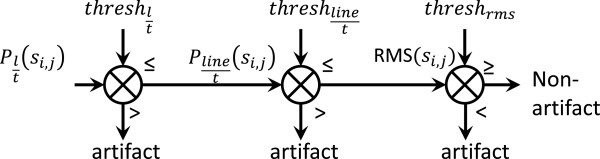
**Schematics of the algorithm for the detection of low quality signals. **The symbols (>) or (<) represent the cases where the feature was expected to be higher or lower than the specified threshold in order to determine if a given signal was labeled as artifact.

The algorithm was applied to a signals set, *S,* composed by signals *s*_*i,j*_ recorded in the rows (*i*) and columns (*j*) of a given electrode array A1 to A3 (*i=[1,2,…,6] and j=[1,2,…,17] or j=[1,2,…,19]* for array A1 depending on the size of the forearm of the subject, and *i=[1,2,…,8]* and *j=[1,2,…,15]* for arrays A2 and A3 in the upper-arm). A schematic of the decision algorithm is presented in Figure 3.

Thresholds *thresh*_*l/t*_, *thresh*_*line/t*_ and *thresh*_*rms*_ were calculated from a subset of *S* composed of signals called reference (*ref)* that satisfied the following two conditions:

(1) $$\text{ref}=\{\begin{array}{l} abs\left(P_{\frac{l}{t}}\left(\text{ref}\right)-\text{median}\left(P_{\frac{l}{t}}\left(S\right)\right)\right)<1.5IQR\left(P_{\frac{l}{t}}\left(S\right)\right)\\ abs\left(P_{\frac{\text{line}}{t}}\left(\text{ref}\right)-\text{median}\left(P_{\frac{\text{line}}{t}}\left(S\right)\right)\right)<1.5IQR\left(P_{\frac{\text{line}}{t}}\left(S\right)\right) \end{array}$$

where IQR represents the Interquartile Range. The median was chosen instead of the mean because of its lower sensitivity to outliers since it considers the highest breakpoint (50%), that is, the smallest percentage of outliers that can cause an estimator to take arbitrary large values [22].

The thresholds were calculated as following:

(2) $$\text{thres}h_{\frac{l}{t}}=k_{1}\left(\text{median}\left(P_{\frac{l}{t}}\left(\text{ref}\right)\right)+1.5\text{IQR}\left(P_{\frac{l}{t}}\left(\text{ref}\right)\right)\right)$$

where the constant *k*_*1*_ was subjected to an optimization criterion in order to improve the performance of the detection method as it is explained in the next section.

(3) $$\text{thres}h_{\frac{\text{line}}{t}}=k_{\text{line}}\left[\text{median}\left(P_{\frac{\text{line}}{t}}\left(\text{ref}\right)\right)+1.5\text{IQR}\left(P_{\frac{\text{line}}{t}}\left(\text{ref}\right)\right)\right]$$

where the constant *k*_*line*_ was subjected to a sensitivity analysis (see Table 1 , section Results) and set to 2.5, as a tradeoff between the capacity of *thresh*_*line/t*_ to identify the highest proportion of signals presenting power-line harmonics and the capacity to correctly identify such signals avoiding the misdetection of good-quality signals. In any case, *thresh*_*line/t*_ was always constrained to 0.85, that is, signals presenting more than 85% of the signal power in the bands related to power-line harmonics were considered as artifacts.

(4) $$\begin{array}{l} \text{thres}h_{\text{rms}}=\text{min}\left\{\mu_{\text{pa}},\mu_{\mathrm{pb}},\mu_{\mathrm{pc}}\right\}\\ \;\;+k_{2}\text{max}\left\{\mathrm{st}d_{\text{pa}},\text{st}d_{\text{pb}},\text{st}d_{\text{pc}}\right\} \end{array}$$

where *μ* and *std* are the average and standard deviation, respectively, of the RMS of the following three neighbor-pairs in the proximity of a given channel *s*_*i,j*_: *p*_*a*_ = [*RMS*_*i* − 1,*j*_, *RMS*_*i* + 1,*j*_] in the longitudinal direction, and *p*_*b*_ = [*RMS*_*i* − 1,*j* − 1_, *RMS*_*i* + 1,*j* + 1_] and *p*_*c*_ = [*RMS*_*i* + 1,*j* − 1_, *RMS*_*i* − 1,*j* + 1_] in the diagonal directions. This third condition distinguished low amplitude signals corresponding to innervations zones or non- active regions (for example R4C3, R5C3 and R6C3 in Figure 2) from isolated low amplitude signals (for example R1C1 in Figure 2) that were considered as artifacts. Hence, the threshold in Eq. 4 takes into account the spatial direction of propagation of Motor Unit Action Potentials. Finally, the constant *k*_*2*_ in Eq. 4 was tuned in order to improve the performance of the method as it is explained in next section.

**Table 1:** **Sensitivity analysis for constant *K***_***line ***_**for the detection of power-line artifacts**

***k***_***line ***_***(2.5)***	**1**	**2**	**3**	**5**	**7**
Acc	99,74	99,79	99,79	99,74	99,79
S	83,33	83,33	83,33	75,00	75,00
SP	99,83	99,87	99,87	99,87	99,91
P	71,43	76,92	76,92	75,00	81,82

*k*_*lin*e_ in Eq. 3 was chosen as 2.5 (presented in parenthesis). Increasing values of *k*_*line*_ reduced the sensitivity (S) of the threshold *thresh*_*line/t*_, whereas decreasing values decreased the precision (P). Variations in these two indexes affected the Accuracy (Acc) and the Specificity (SP). *k*_*line*_ = 2.5 is a good compromise between S and P.

#### Training and validation

Constant values of *k*_*1*_ and *k*_*2*_ on eq. 2 and eq. 4, allowed to increase the performance of the detection method, especially its sensitivity (or the capacity of the method to identify the highest proportion of low-quality signals) and the precision (or the capacity to correctly identify low-quality signals avoiding the misdetection of good signals as low-quality). Thus, the performance of the method was measured in terms of Sensitivity (S), Specificity (SP), Precision (P) and Accuracy (Acc) as [23]:

(5) $$\begin{array}{l} S=\frac{\text{TP}}{\text{TP}+\mathrm{FN}}\cdot \mathrm{SP}=\frac{\text{TN}}{\text{TN}+\text{FP}}\;P\\ \;\;=\frac{\text{TP}}{\text{TP}+\text{FP}}\cdot \text{Acc}=\frac{\text{TP}+\text{TN}}{\text{TP}+\text{FP}+\text{TN}+\text{FN}} \end{array}$$

where TP and TN is the number of channels correctly identified as low and good-quality signals respectively, FN is the number of low-quality signals not identified by the algorithm and FP is the number of good-quality signals identified as low-quality.

Signals were visually classified as low-quality or not by three experts based on the observation of similarity between different channels and on the examination of baseline fluctuations or periodicity patterns (related to movement artifacts and power-line interference respectively, see Figure 2a as an example). Two databases for training and validation were obtained, each composed of 20 signal sets *S* selected from different contractions, effort levels and arrays 1 to 3. Fleiss’ Kappa index [24] was used to measure agreement between experts, scoring 82.63% and 86.19% for the training and validation databases respectively and indicating an “almost perfect agreement”. The information of the three experts was combined by obtaining the majority vote in each case (i.e. the statistical mode of the three opinions) in order to obtain a binary classification-label as artifact/non artifacts for each single-channel in the set *S*.

Optimal values for *k*_*1*_ and *k*_*2*_were tuned according to the following criteria: 1) by Receiver Operating Characteristic (ROC) curves (S vs. 1-SP), widely used in signal detection theory and clinical diagnostics [25] and, 2) by Precision-Recall representations (P vs. SP), which are commonly used in machine learning [26]. Both methods assessed the accuracy of the prediction (or outcome) of the described method based on its Sensitivity, Specificity and Precision. The optimal classifier was found as a tradeoff between hit rates and false alarm rates. In the first case the optimal can be found as the minimum distance between the curve (S vs. 1-SP) and the point [0, 1], and in the second case, as the minimum distance between curve (P vs. SP) and the point [1, 1].

### Segmentation of HD-EMG maps

Areas corresponding to electrodes lying over an active region of a muscle or a set of muscles can change between subjects. Therefore, it was useful to extract a delimited region related to each muscle of interest before averaging between subjects, in order to finally obtain a general activation map for the different tasks. Thus, an algorithm for the segmentation of active regions was proposed in this study. An activation map *I* in the 2D space was calculated from HD-EMG signals as:

(6) $$I_{i,j}=\frac{1}{M}\sum_{m=1}^{M}\left(\frac{1}{N}\sum_{n=1}^{N}s_{i,j}^{2}\left(n\right)\right)=\frac{1}{M}\sum_{m=1}^{M}\text{RMS}\left(s_{i,j}\right)$$

where *s* is an EMG signal in the channel located at the position *i, j* of the 2D array (as explained before), N is the total number of samples in an epoch of 500 ms and the RMS value was averaged in M=6 non overlapped epochs corresponding to three seconds of signal. Prior to the calculation of RMS, the signals were filtered between 12 and 350 Hz with a 4th order Butterworth filter in forward and backward direction in order to correct for phase distortion following SENIAM recommendations for the processing of surface EMG signals [27]. RMS values corresponding to signals previously identified as artifacts were replaced by triangle-based cubic interpolation based on Delaunay Triangulation for surface fitting proposed in [28].

Each channel in the map can be considered as a pixel located at the positions *i* and *j* of the electrode array whose intensity is given by *I*_*i,j*_. In other words, the map *I* can be thought of three dimensions where the intensity values represent elevations as in a topographical map. Intensity levels in the maps correspond to the activation level of a muscle (or a set of muscles) during the development of a specific task.

The activation map was segmented by applying an h-dome transform *D*_*h*_*(I)* over the image *I*, as proposed by L. Vincent in [17]. Mathematically, the transformation is defined as:

(7) $$D_{h}\left(I\right)=I-\rho_{I}\left(J\right)=I-\rho_{I}\left(I-h\right)$$

where the operator *ρ*_*I*_ stand for morphological reconstruction [29], and *J* is derived from *I* by subtracting a constant value *h.* This transformation preserved all the domes above the height *h*, including those that contain various local maxima. In the case of activation maps, such local maxima could correspond to local variations of the amplitude of the 2D maps due to distinct contact impedances in the various electrodes. Additionally, a morphological opening (γ) was applied to the resulting image *D*_*h*_ in order to avoid the segmentation of isolated peaks, small in area, and which corresponded to pixels with an amplitude slightly higher than that of the surrounding channels. This was the case in “flat” maps (particularly at low-effort levels) where the activation is mainly reflected on the synergistic muscles with levels comparable to background noise and where only marginal activation can be observed in antagonist muscles. Therefore, the final segmented image *D’*_*h*_ was obtained as:

(8) $$D{'}_{h}=\gamma \left(D_{h}\right)=D_{h}\circ b=\left(D_{h}⊖b\right)⊕b$$

where *b*, the structuring element, is a disc of radius 1 and ⊖ and ⊕ are the operators for dilation and erosion respectively [29].

### Average HD-EMG maps

Average maps for the 12 subjects who participated in the study were obtained by averaging individual segmented maps, in order to obtain useful information related to muscle co-activation pattern in terms of intensity values and its spatial distribution during different kind of tasks and levels of effort. Considering that upper-limb dimensions, specifically circumference and length, are different for every subject, it was necessary to normalize and interpolate the image in the 2-D space so that results could be comparable among subjects allowing the calculation of an average map for the population. In the case of arrays A2 and A3 (biceps and triceps), the zero of the coordinate system was defined to correspond to landmarks defined by SEMIAM project [20]. In the case of forearm muscles, the origin of the *x-axis* laid in the intersection of the line connecting the origin and insertion of each muscle (Anconeus, Brachioradialis or Pronator Teres) and an arch traced around the forearm, 2 cm below the elbow crease which in turn was the zero of the *y-axis.* Values in the *x* dimension were normalized with respect to the circumferences of the different arm segments: proximal forearm for array A1, distal upper-arm for array A2 and proximal upper-arm for array A3, and values in the *y* dimension were normalized with respect to the muscle length as described in the protocol description. In order to obtain individual maps with the same (*i, j*) coordinates for the calculation of population’s average map, RMS values in the 2-D space were re-sampled by cubic splines interpolation in the *x* and *y* directions, considering units relative to the total length and total circumference of the arm segment as explained in the previous paragraph.

Intensity of the maps was parameterized by the average RMS value (RMS_av-HD_) for the segmented area. In addition, spatial distribution of the maps was parameterized according to the median of its projection over the *x* and *y* axes, that is, *x=μ*_*x*_ or *y= μ*_*y*_ where such projection was divided into two regions of equal area, as following:

(9) $$\begin{array}{l} \sum_{k=1}^{\mu_{\text{dim}}}Q_{k}^{\text{dim}}=\sum_{k=\mu_{\text{dim}}}^{M}Q_{k}^{\dim}=\frac{1}{2}\sum_{k=1}^{M}Q_{k}^{\text{dim}}\\ \text{where}\;Q_{k}^{\text{dim}}=\max(D_{h}^{'},dim) \end{array}$$

where *Q*_*k*_^*dim*^ is the value of the projection of the maximum of the segmented map *D’*_*h*_ at the *k*^*th*^ coordinate along the dimension *dim= x* or *dim=y* and *μ*_*dim*_ corresponds to the median of the projection *Q*_*k*_^*dim*^*.* The median of the projections permitted to evaluate spatial shifts along the *x* and *y-*axes of the maps, both of them associated with changes in effort levels or even with different tasks.

On the other hand, data similar to that obtained with bipolar electrodes was extracted by selecting two monopolar channels for each muscle in the 2D arrays: the first electrode corresponded to the one located at the origin of the coordinate system (i.e. reference in SENIAM recommendations [20]) as described previously for each muscle, and the second one was located 10 mm apart in the direction of the muscular fibers. For each muscle, a single differential signal was obtained from these two channels and its corresponding RMS_av-bip_ value was calculated at the same time-interval as in the case of HD-EMG maps. The variables RMS_av-bip_ and RMS_av-HD_ (from bipolar or HD-EMG configurations) were used in the identification of tasks and their performances were compared as later explained.

### Statistical analysis

The statistical analysis was intended to assess differences between information extracted from single bipolar signals or from HD-EMG maps and also to analyze differences due to type of task and effort level. Such analysis was based in the variables determined in the previous section, that is, RMS_av-bip_ and RMS_av-HD_ both of them related to the signal power, and μ_x_ and μ_y_ associated with the spatial distribution of the maps.

Factors considered in the statistical analysis were: 1) the type of electrode (bipolar or HD-EMG), 2) The type of task, that is, flexion, extension, supination and pronation, and contraction level (10%, 30% and 50% MVC), and 3) Muscle (i.e. biceps, triceps, brachioradialis, anconeus and pronator teres).

Differences between RMS_av-bip_ and RMS_av-HD_ were evaluated with the non-parametric Friedman test in two-way layout. In order to avoid differences between the type of recording (bipolar and monopolar), both variables in each muscle were normalized with respect to the mean value obtained in all tasks and contraction levels. Data corresponding to different tasks and effort levels were pooled together by considering *blocks* with replication of cells of the factor muscle according to the procedure described in [30] and implemented in the statistical toolbox of MATLAB®.

On the other hand, the capacity of the extracted variables (RMS_av-bip_ or RMS_av-HD)_ for the identification of tasks and/or effort levels was evaluated by classifying the data into different groups based on linear discriminant analysis [31] and cross-validation with the Leaving One Out Method [32]. Data was classified into four or twelve groups corresponding to type of task or to type of task and effort level respectively. In the former case, samples corresponding to the three levels of effort were pooled together for each type of task. The overall classification performance was obtained in terms of Accuracy, Sensitivity, Specificity and Precision as described in [23] and (Eq. 5). For this analysis TP were data samples well classified into any of the classes, FN corresponded to the number of missing samples in any of the classes, that is, samples belonging to a given class but that were classified in another, TN were not misclassified samples, and FP were samples misclassified in any of the classes.

In addition, changes in the spatial distribution of the maps due to type of task and effort level were analyzed with a non-parametric repeated measures design based on the Friedman test. In this case, variations of the variables *μ*_*x*_ and *μ*_*y*_ were evaluated in 12 different measures corresponding to four types of task by three levels of effort each. A Bonferroni correction was applied in order to take into account the multiple comparisons.

Finally, pair-wise comparisons were analyzed trough non-parametric Wilcoxon signed rank test.

Statistical significance was set to p=0.05. In the Friedman test, *χ*^2^ statistics was considered significant for *χ*^2^ (d.o.f=1)> 3.84 for one degree of freedom (d.o.f = 2 types of electrode −1) and for *χ*^2^ (d.o.f=11)> 27.28 for eleven degrees of freedom (d.o.f=12 measures −1) after the Bonferroni correction.

## Results

### Detection of low-quality signals

Sensitivity analysis in the training database for constant *k*_*line*_ in Eq. 3 is presented in Table 1. Higher values of *k*_*line*_ increased the number of TP and FP affecting both, the sensitivity and the precision of the detection. As it can be observed on Table 1, *k*_*line*_ between 2 and 3 is a good compromise between sensitivity and precision and it can be confirmed from the accuracy and specificity, where the highest values were obtained. For values lower than 2, precision decreases whereas for values higher than 3, sensitivity decreases.

**Figure 4:**
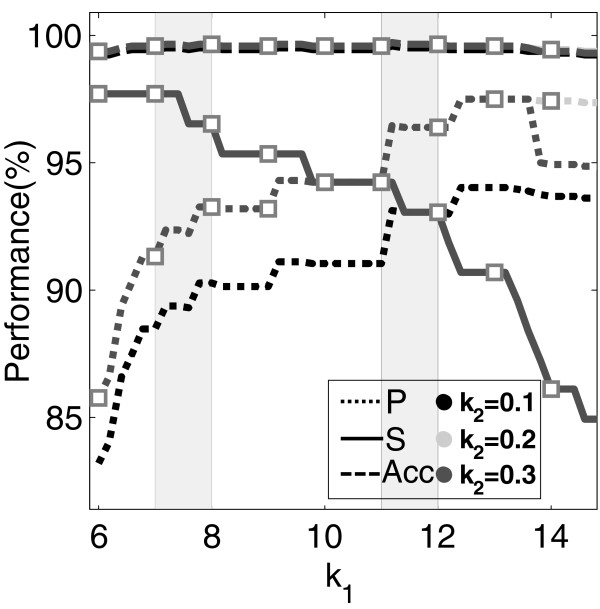
**Sensitivity analysis for *k***_***1 ***_**and *k***_***2 ***_**in the training database. **Constant *k*_*1*_ is represented along the x-axis. Curves (in shades of gray) are shown for *k*_*2*_. Higher values of *k*_*1*_ increased P (in dot lines) but decreased S (in solid lines). Acc (dash lines) remained almost constant for different values of *k*_*1*_ and *k*_*2*_. Optimal values for *k*_*1*_ were found as a compromise between S and SP and the obtained ranges are highlighted in gray-shadowed areas. Performance indexes corresponding to the selected *k*_*2*_=0.2 are shown in squares.

Performance indexes for the detection of artifacts in the training and validation databases using ROC and PR are shown in Table 2. Although results were similar when comparing both criteria, the precision was higher for the PR approach at the expense of slightly lower sensitivity because of the introduction of a number of FN. The sensitivity was higher when considering the ROC criterion but this led to an increase in the number of FP (as compared to PR). The specificity was always very high (above 99%) because the number of non-artifact signals is much higher than the number of low-quality signals. A sensitivity analysis for *k*_*1*_ and *k*_*2*_ in the training database is presented in Figure 4. Different values of *k*_*2*_ produced the same sensitivity for increasing values of *k*_*1*_ whereas the precision varied at high values of *k*_*1*_ (because of the inclusion of FP). Precision and sensitivity increased and decreased respectively for increasing values of *k*_*1*_. The values adopted for *k*_*1*_ and *k*_*2*_ in this work represent a good tradeoff between sensitivity, specificity and precision.

**Table 2:** **Optimum values of *k1 *and *k2 *(Eq.2 and Eq.4) and their performance indexes**

		***(k***_***1, ***_***k***_***2***_***)***	**Acc(%)**	**S(%)**	**SP(%)**	**P(%)**
**Training**	ROC	(7.1, 0.2)	99.61	97.67	99.69	92.31
	PR	(11.2, 0.2)	99.66	94.19	99.87	96.43
**Validation**	ROC	(7.1, 0.2)	99.40	97.94	99.46	88.78
	PR	(11.2, 0.2)	99.48	92.78	99.77	94.73

Performance measures for combinations of k_1_ and k_2_: Accuracy (A), Sensitivity (S), Specificity (SP) and Precision (P) for the training (20 sets) and validation (20 sets) databases. The two methods proposed for outlier detection are presented (ROC, Receiver Operating characteristics and PR, Precision-Recall).

Although high Sensitivity (S) and Specificity (SP) were desired, a lower number of FP became important when considering the next step of the analysis where RMS values of artifacts were interpolated from neighbor channels. If too many channels in the neighborhood were wrongly labeled as “artifacts” (i.e. too many FP), then the interpolation process was not possible. Because of the lower Precision of the algorithm with the ROC criterion (Table 2), constants *k*_*1*_ and *k*_*2*_ were finally selected according to optimization results obtained by Precision- Recall.

On the other hand, the algorithm for artifact detection had low computational complexity. The execution time of the algorithm, (mean and standard deviation for joint training and validation databases), was 201.2±12.85 ms, [min: 187.5 ms, max 234.4 ms] per signal set on a 2.13 GHz Intel® Core2™ processor. Each set had a total duration of 3s and was composed by a different number of channels between 109 and 120 channels.

### Segmentation

**Figure 5:**
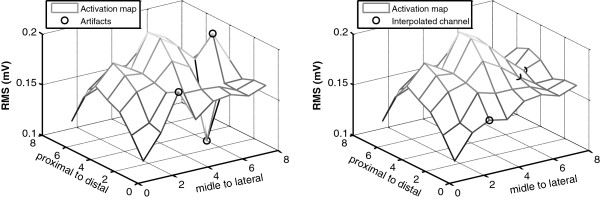
**Substitution of RMS values due to artifact signals. **RMS values of artifact channels are replaced by fitting the surface described by nearby non-artifact channels. *Left*. Sharp peaks caused by artifacts are marked with a circle. *Right*. Fitted surface obtained after replacing RMS values detected as artifacts (marked with a circle). Such points were replaced by fitting the surface described by non-artifact neighboring channels.

**Figure 6:**
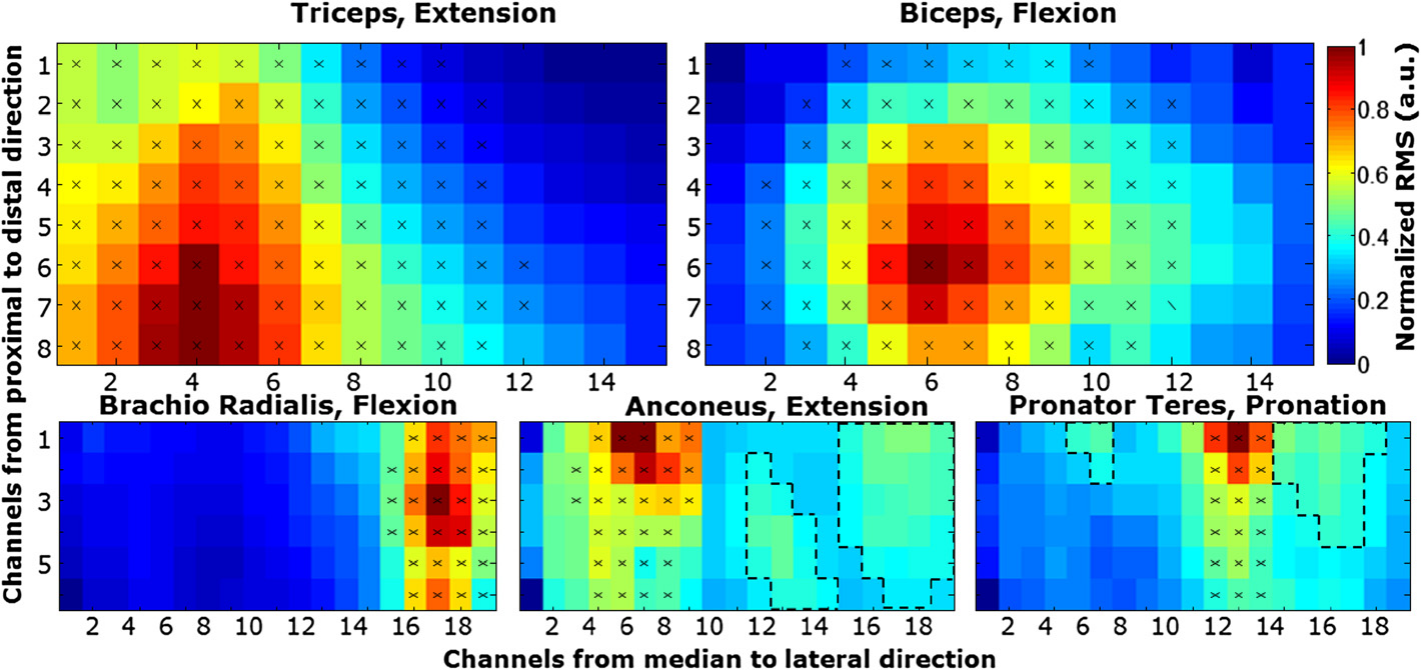
**Example of segmentation of HD- EMG Maps from the right arm of subject 5: Triceps (top left), Biceps (top right), Brachio Radialis (bottom left), Anconeus (bottom middle), and Pronator Teres (bottom right). **Maps corresponding to 50% MVC in exercises associated with the main function of each muscle are presented. Final segmented regions are presented with crosses. Regions limited by dash lines were also segmented and considered as belonging to neighboring muscles and were not taken into account to obtain the average map for the 12 subjects.

An example of the surface obtained after the triangle-based cubic interpolation of channels identified as artifacts can be observed in Figure 5. Additionally, an example of the segmentation of different maps obtained for flexion, extension, pronation and supination in the five muscles is presented in Figure 6. The shape of the segmented area depended on the intensity of the peaks.

The segmentation produced intensity maps for each muscle and subject, and allowed the calculation of average HD-EMG maps representative for the 12 subjects in the study.

### *Bipolar* vs. *High-density EMG signals*

Significant differences were observed for the two-way Friedman test between RMS_av-bip_ and RMS_av-HD_ using the factor *muscle* as blocking factor (*χ*^2^[1] = 16.55, p<0.001). Thus, significant differences were observed when characterizing the different tasks and effort levels with information extracted from one or the other type of sensor.

**Figure 7:**
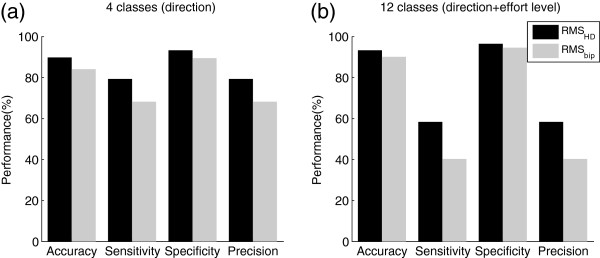
**Classification performance using the feature RMS**_**av-HD **_**(in black) extracted from HD-EMG maps or RMS**_**av-bip **_**(in gray) extracted from single bipolar EMG signals. a**) Four classes corresponding to type of task (flexion, extension, pronation and supination) or **b**) Twelve classes corresponding to type of task and effort level (10%, 30% or 50% MVC).

Results for the overall classification into four or twelve groups are presented in Figure 7 for the variables RMS_av-bip_ and RMS_av-HD_.

### Average HD-EMG Maps

**Figure 8:**
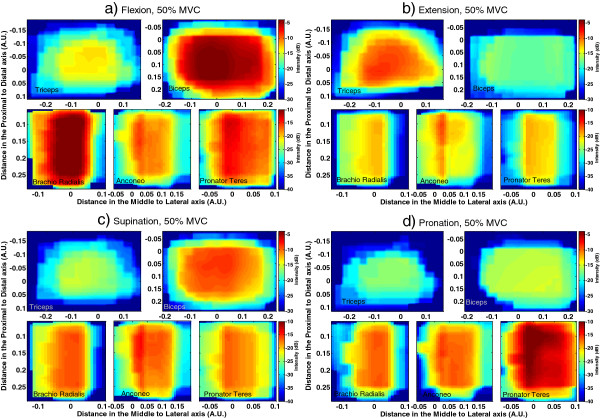
**Average HD-EMG maps across subjects in the five assessed muscles: Triceps (top left), Biceps (top right), Brachioradialis (bottom left), Anconeus (bottom middle) and Pronator Teres (bottom right). **Maps are displayed in dB. The color scale for biceps and triceps is presented at the top of each figure and the color scale for brachioradialis, anconeus and pronator teres is presented at the bottom. **a**) Flexion at 50% MVC, **b**) Extension at 50% MVC, **c**) Supination at 50% MVC. **d**) Pronation at 50% MVC.

Average activation maps for the 12 subjects at 10, 30 and 50% MVC for each of the four tasks under study were obtained by averaging the individual maps of each subject. Results on average maps at 50% MVC are displayed in Figure 8. It is possible to observe different muscle co-activation patterns resulting from muscle interactions when performing a given task. Variability between subjects was measured through the standard deviation divided by the intensity for each pixel of the average map. Results are presented in Table 3.

**Table 3:** Variability between the 12 individual maps associated with the average HD-EMG maps

	***10%***	***30%***	***50%***
Biceps (mean, [min max])	0.085, [0.07, 0.11]	0.18, [0.16, 0.2]	0.29, [0.23, 0.39]
Triceps (mean, [min max])	0.13, [0.084, 0.23]	0.15, [0.089, 0.2]	0.24, [0.11, 0.36]
Brachio Radialis (mean, [min max])	0.14, [0.063, 0.25]	0.16, [0.13, 0.21]	0.23, [0.17, 0.26]
Anconeus (mean, [min max])	0.13, [0.11, 0.14]	0.18, [0.12, 0.22]	0.2, [0.15, 0.23]
Pronator Teres (mean, [min max])	0.12, [0.063, 0.2]	0.16, [0.11, 0.2]	0.22, [0.15, 0.29]

Variability was measured through the standard deviation divided by the intensity for each pixel of the average map. Mean variability is shown by averaging the variability values over the segmented region for the four tasks. Results are presented as mean for 12 subjects including minimum and maximum values are. This average was carried out for each muscle and each level of effort.

**Figure 9:**
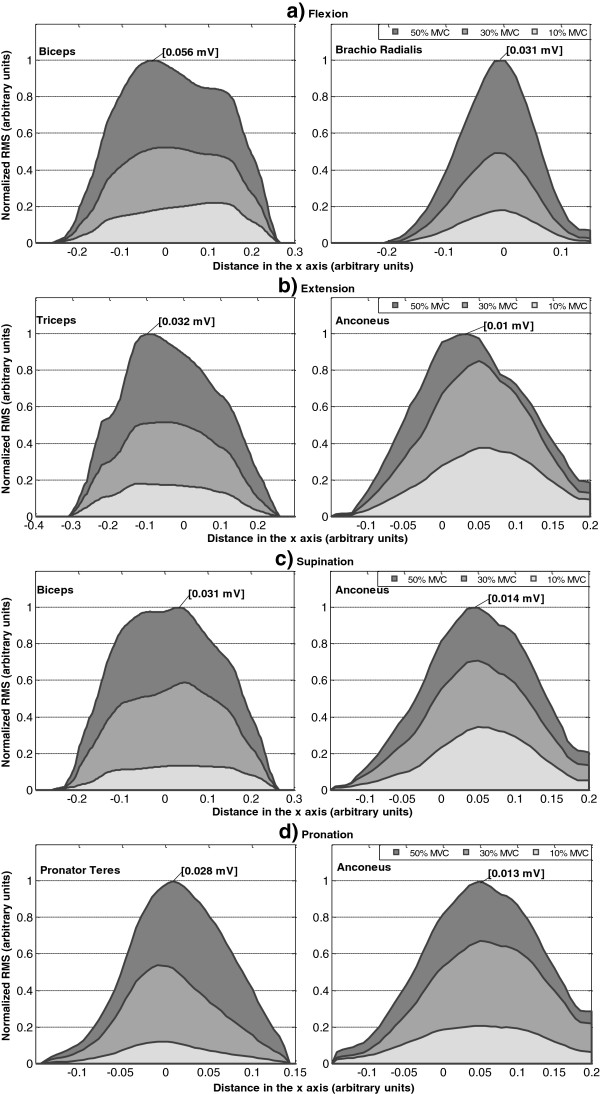
**Projections *Q***_***k***_^***x ***^**from the two most active muscles for each kind of task. **Curves are normalized with respect to the maximum intensity reached at 50% MVC (showed with a label in each plot). **a)** Flexion in Biceps and Brachio Radialis, **b)** Extension in Triceps and Anconeus. **c)** Supination in Biceps and Anconeus, and **d)** Pronation in Pronator Teres and Anconeus.

In addition to the average maps at 50% MVC, their projections *Q*_*k*_^*x*^ at 10%, 30% and 50% MVC for the two most active muscles in each type of task are presented in Figure 9. Each projection was normalized with respect to the maximum value reached at 50% MVC in order to observe changes of the intensity as a function of effort level and to compare among muscles and/or tasks. Changes observed are summarized as following:

• Flexion: Intensity decreased with decreasing levels of effort (from 50 to 10% MVC) in Biceps and Brachioradialis (p<0.0005), preserving a similar proportion in both muscles (Figure 9a).

• Extension: Intensity in Triceps decayed proportionally from 50% to 10% MVC (p<0.0005), but not in the Anconeus, where the maximum of the distribution was significantly different between 10% and 30% MVC (p<0.009) but not between 30% and 50% MVC (Figure 9b).

• Supination: Intensity decreased from 50 to 10% MVC in Biceps and Anconeus (p<0.0005) but not in the same proportion in both muscles. Changes in the activation of the Anconeus were proportionally higher at 10% and 30% MVC when compared to changes in biceps, (p<0.003), although the former continued being the most active muscle in the contraction (Figure 9c).

• Pronation: Intensity levels in Pronator Teres and Anconeus decreased similarly from 50 to 10% MVC. However, the normalized levels for Anconeus at 10%MVC were higher than those for Pronator Teres (p<0.001) showing that the contribution of the former was higher at lower levels of contraction. (Figure 9d).

The identification of movement intention by muscle co-activation is usual in pattern recognition approaches [33]. Although such co-activation pattern has already been assessed with bipolar electrodes, performance of the identification of tasks and even of the intended effort level were improved when considering HD-EMG maps from the upper-arm and forearm (see Figure 7). Additionally, differences in the co-activation of muscles were also reflected in the spatial distribution of the maps. Results for the repeated measures Friedman test for the spatial distribution variables *μ*_*x*_ and *μ*_*y*_ extracted from HD-EMG maps are presented in Table 4. It is possible to observe that the obtained *χ*^2^ values were significant for four of the five analyzed muscles confirming possible changes in the spatial distribution with different tasks and/or effort levels.

**Table 4:** **Results for the repeated measured friedman test for the variables *μ***_**x **_and ***μ***_**y **_**extracted from HD_EMG**

	***χ***^***2***^[11]**,**[12]		***p***	
	***μ***_***x***_	***μ***_***y***_	***μ***_***x***_	***μ***_***y***_
Biceps	47.84*	28.94*	<0.001*	<0.002*
Triceps	33.18*	42.98*	<0.001*	<0.001*
Brachioradialis	8.99	23.52	n.s.	n.s.
Anconeus	8.68	27.43*	n.s.	<0.004*
Pronator Teres	45.08*	31.96*	<0.001*	<0.001*

The table presents the *X*^2^[11,12] statistics for 11 d.o.f. and N=12 samples, and its associated significance value P for the five muscles. Statistical differences are marked with (*).

Furthermore, pair-wise comparisons between spatial-distribution variables obtained for different effort levels of the same task on a given muscle in Figure 9 were assessed by applying a Wilcoxon signed rank test to the medians. For example, μ_x_ in the Biceps, shifted to the left with increasing levels of contraction during flexion (p<0.02) and during supination (p<0.05). Such shifts were likely due to differences in the activation of the two heads of this muscle. Additionally, significant shifts were also found for the Pronator Teres during pronation (p<0.05).

**Figure 10:**
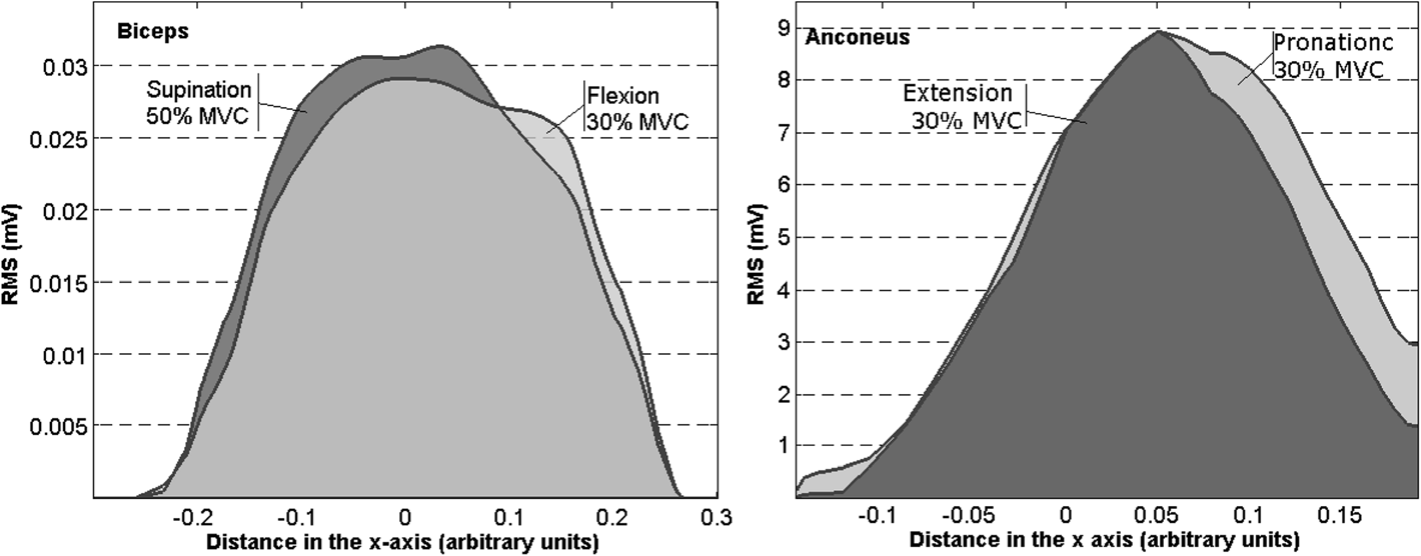
**Projections *Q***_***k***_^***x ***^**for Biceps during Supination at 50% MVC and Flexion at 30% MVC (left) and Anconeus during extension at 30% MVC and Pronation at 30% MVC (right). **Although the maximum and the area under each curve were similar for different exercises (p.n.s), their projection shifted laterally depending on the exercise kind.

Finally, interesting results were obtained when comparing spatial distributions between different kinds of task for the same muscle. Intensity levels can be very similar, with no significant differences in the absolute maxima of the projections, but differences were found in the spatial distribution along the x-axis. An example is presented for Biceps during supination at 50% MVC and flexion at 30% MVC in Figure 10: The maxima of the projections *Q*_*k*_^*x*^ were not significantly different (p.n.s) but their spatial distribution differed (i.e. different median *μ*_*x*_, p<0.04). This effect was also observed in the Anconeus (Figure 10.) when considering an extension and a pronation, both at 30% MVC (p<0.05 for the location of *μ*_*x*_). Therefore, even when in these two examples the same muscle is active with similar levels of intensity, only the spatial distribution permitted to observe differences between tasks.

## Discussion and conclusions

The main objective of the study was to extract information from HD-EMG maps that could be associated with four tasks at the elbow joint (forearm pronation and supination, and elbow flexion and extension) at different effort levels. This objective has been reached using 2D arrays of electrodes on five muscles in the upper-arm and forearm. It was shown no only that the average signal power extracted from HD-EMG maps may improve the differentiation of tasks and effort levels but also that the spatial distribution of the maps differed between tasks. Variables related with the spatial distribution of the intensity may complement the information provided by the amplitude of the signals in the identification of motion intention. Additionally, average HD-EMG maps for the four types of tasks and for the group of 12 voluntary subjects were obtained.

### Detection of low-quality signals

Due to the high number of sEMG channels, an algorithm for the automatic detection of low quality signals was developed. In this study, the artifactual channels were removed and interpolated based on RMS values of neighbor channels in the HD-EMG maps.

As the number of channels affected by artifacts is usually much lower than the number of non- artifact channels, the number of TN is usually high, so the accuracy and especially, the specificity, is in general very high (>99%). In this study, the percentage of low quality signals was between 0 and 13% of the total number of channels of each set in the training database. For this reason, the ROC method, which was intended to compromise between specificity and sensitivity, provided an overestimated value of the former (the latter was always very high as it was explained above) at the expense of reducing the precision of the algorithm. On the other hand, PR method maximized the sensitivity while preserving the precision. Considering that both, the specificity and the accuracy were always very high, the use of PR was more convenient in our case since we were dealing with offline detection and interpolation of artifacts. In addition, the accuracy was slightly higher with the PR method. Other applications, especially those intended for online detection, should consider the ROC approach in order to increase the sensitivity as much as possible.

In addition, the sensitivity analysis for the constants *k*_*line*_, *k*_*1*_ and *k*_*2*_ in Tables 1 and 2 showed that the selected values represented a good compromise between the Precision and Sensitivity of the detection.

Different approaches have also been suggested for the detection of low quality signals. In [16], a non-supervised method based on local distance-based outlier factor was proposed for HD-EMG signals recorded from the same muscles and electrodes arrays. That method did not required a training process and successfully detected low-quality signals with an average Accuracy of 91.9%, Sensitivity of 96.9% and Specificity of 96.4%. If the latter index is considered (refer to *SP* in Eq. 5), it is possible that such method was prone to the inclusion of FP, given that the number of TN is always very high as explained before. This condition was not desired in our study because intensity values associated with low quality signals were later replaced in the maps based on neighbor channels, and if those were wrongly identified as artifacts (FP), the interpolation for the replacement was not possible. Thus, another method intended to minimize the number of FP while preserving the accuracy of the detection was proposed in the present work. Moreover, the method in [16] did not took into account information provided by neighbor channels as in the algorithm described here.

A different study by Gronlund et al. in [15] assumed unimodal distribution of the amplitude of the signals and therefore it was not applicable to cases where the multichannel recording involved various regions with different levels of activity (as in Figure 2) or even with no activity at all. The latter corresponded to regions localized far away from the main activation areas or to muscles marginally active during the contraction (an example can be observed in Figure 6 bottom-left for the signals recorded in array A1-forearm during flexion). In contrast, the algorithm proposed in the present work labeled a signal as an artifact based on the amplitude information of 6 neighboring channels (Eq. 4), avoiding general assumptions on the distribution of the potentials recorded in the array as proposed in [15].

Finally, the algorithm reached very high values for the performance indexes in the validation dataset (see Table 2). In addition, the channels identified as artifacts were correctly interpolated from the information of neighboring non-artifact channels even at the edges of the map (see Figure 5), which provided a smooth surface for subsequent stages of the analysis. Therefore, both the proposed methodology and features showed a very good performance for the detection and off-line replacement of low-quality signals.

### Segmentation of HD-EMG maps

An automatic algorithm for the segmentation of active zones was proposed. This segmentation allowed the calculation of average HD-EMG maps for the population of 12 subjects by determining the ranges of active zones in the *x* and *y* axes relative to upper-limb circumference and muscle length respectively, and referred to the electrode location recommended for sEMG recording on the analyzed muscles.

**Figure 11:**
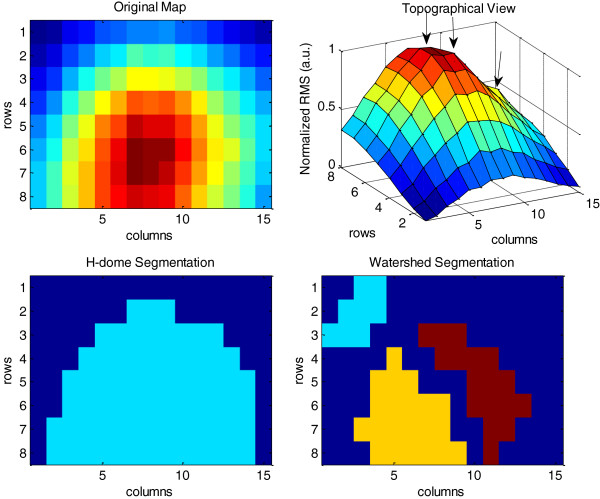
**Segmentation of HD-EMG map from biceps of subject 2 during flexion at 10% MVC. **The original map and its topographical view is presented at the top of the figure (left and right respectively). The segmentation obtained by h-dome transform is presented at bottom-left. The active zone in light blue is correctly isolated from the background image (displayed in dark blue). The watershed segmentation obtained 3 different zones: light blue, yellow and dark red (bottom-right) as consequence of multiple regional maxima indicated by arrows in the topographical view of the map. The final result of the segmentation is obtained after discarding those pixels whose intensity value is below 70% of the maximum intensity in each of the segmented zones. Such pixels correspond to the background (dark blue).

Other methods have been previously proposed for the segmentation of activation maps. Particularly Vieira et al. in [9] proposed a method based on watershed for assessing muscle compartmentalization. Such segmentation was aimed at the extraction of zones associated with local variations in the level of neuromuscular activity in the same muscle and it was successfully applied with this objective on the gastrocnemius. However, we were interested in obtaining activation maps for different muscles, each of them associated with different types of contractions and levels of effort. This purpose required the isolation of muscle activity from background in individual maps before their average. Thus, the segmentation algorithm had to extract global active regions, even if those were composed by several regional maxima. This last condition would lead to an over-segmentation in terms of the purpose of our study when using Watershed techniques (see Figure 11). On the other hand, h-dome was applicable straightforward without need of previous equalization or transformation of the original image and was not sensitive to regional maxima [28]. For this reason the *h-dome* transformation was preferred in this work.

Furthermore, it is important to note that maps segmentation was not the aim of this study neither the determination of anatomical muscle regions but was an intermediate step in order to obtain average maps. It is known that potentials’ amplitude diffuses across skin surface, so the actual size of active muscle regions might be overestimated by the segmentation proposed. In spite of this, the segmentation permitted the extraction of surface areas corresponding to different muscles in individual maps and also permitted to focus on the regions of major activation, avoiding other active neighboring areas that could be more affected by the activity of nearby muscles (see Figure 6). By averaging individual segmented maps, it was possible to obtain representative HD-EMG maps associated with the activation of each muscle during the different tasks and effort levels. In this sense, the segmentation worked properly.

### Average HD-EMG maps

Variability in the levels of intensity between subjects with respect to the average HD-EMG maps (Table 3) was found to be low enough to consider such maps as representative for the 12 subjects. The obtained differences were higher for higher levels of contraction (between 20% and 29% in the four tasks at 50% MVC) and being less than 12% for contractions at 10% MVC (see Table 3). In the average activation maps depicted in Figure 8, it is possible to observe changes in the activation pattern corresponding to different tasks. Such differences were confirmed with the classification by LDA shown in Figure 7. Consequently, results concerning the spatial distribution and the levels of intensity from average maps can be considered as globally associated with the muscle function and to the activation pattern of the subjects in the study.

On the other hand, the classification performance was higher for the average power extracted from HD-EMG maps (RMS_av-HD_) than from bipolar electrodes (RMS_av-bip_) either for four or twelve classes, especially when considering the precision and sensitivity of the classification. Therefore it is possible to conclude that information extracted from the amplitude of signals recorded in high-dimensional configuration has more power to differentiate between tasks and even effort levels than single bipolar signals. With this respect, a recent study by Tkach et al. [33] based on information extracted from bipolar signals showed that the classification accuracy for the identification of motor tasks worsens when considering distinct strengths of the same motion. Substantial drops were observed when training and testing the classifier with data of mixed high and low effort levels, obtaining a maximal accuracy of ~80% for the classification into the four tasks described in the present work. In our case, an overall classification accuracy of approximately 90% for 4 motor tasks with mixed data from very-low to medium-high effort levels (10%, 30% and 50% MVC) was obtained. What is more, classification accuracy for 12 classes was also in the order of 90%. However, in both cases the precision and sensitivity were not very high, thus additional data transformations or other features are necessary in order to improve the identification performance. In this sense, the Friedman test showed that variables related to the spatial distribution of the maps (*μ*_*x*_ and *μ*_*y*_, see Table 4) may also assist in the discrimination between types of tasks and effort levels.

In addition, Tkach et al. also found that classification accuracy dropped when bipolar electrodes shifted by15 mm [33]. Such shifts could be due to relative movements between the recording electrodes and the skin or because of errors when positioning the sensors, for example in different days. Thus another advantage of HD-EMG maps relies on the contact redundancy implied by the recording of a number of signals over a large surface of the muscle, as well as in the possibility of extracting features associated with spatial-changes induced by the central nervous system in the control of the muscles [11]. All of this makes HD-EMG maps more robust to errors introduced by contact artifacts and by the relative location of the electrodes with respect to the origin of the potentials, especially in contractions involving joint movement or sensor repositioning.

When analyzing variables associated with the activation maps for the 12 subjects, it was possible to observe differences in the co-activation pattern of the muscles according to the kind of task and the effort level. For example during flexion at 50% MVC (see Figure 6), the Biceps, and the Brachioradialis were the most active muscles (as expected) but there was also an important activation of the Anconeus and of the Pronator Teres likely to stabilize the elbow joint and to compensate for the supination action of the biceps. The extension at 50% MVC was mainly produced by the Triceps and the Anconeus but the other two analyzed muscles in the forearm were also active. All selected muscles but Triceps were involved during Supination at 50% MVC with similar intensity levels among them. Finally, during Pronation at 50% MVC, naturally the most active muscle was the Pronator Teres but both the Anconeus and the Brachioradialis were also active while the muscles of the upper-arm appeared not to be active during the contraction. Additionally, when considering activation patterns for contractions at 30% and 10% MVC it was found that the levels of intensity did not decreased proportionally with effort level in all of the muscles, showing changes in the load-sharing of the involved muscles.

Differences in the average maps between 10%, 30% and 50% MVC were not only related to the levels of intensity but also to its spatial distribution. It was possible to observe differences in their projection over the *x*-axis (see Figure 9 and 10 and Table 4) for different effort levels depending on the task. These variations corresponded to shifts in the lateral to medial axis when the level of effort changed from 10% to 50% MVC. Furthermore, similar values of intensity were obtained for different kind of tasks in some muscles and differences were only found in the spatial distributions of the maps.

For all of this, HD-EMG maps instead of single bipolar signals, and variables related to maps intensity and spatial distribution might be useful in applications where identification of movement intention is needed: for example in robotic-aided therapies focused on the improvement of muscle coordination where interaction between patient and machine is involved and where the robot has to be able to sense patient’s intention [34]. Additionally, other applications implying proportional control for devices like powered- prostheses or orthoses could benefit from information provided by HD-EMG maps regarding not only the task but also its strength.

## Abbreviations

Acc: Accuracy; DRL: Diven Right Leg; EMG: Electromyography; FN: False negatives; FP: False Positives; HD-EMG: High Density Surface Electromyography; MVC: Maximal Voluntary Contraction; P: Precision; PSD: Power Spectral Density; PR: Precision- Recall representation; RMS: Root Mean Square; ROC: Receiver Operating Characteristics; S: Sensitivity; SP: Specificity; TN: True Negatives; TP: True Positives.

## Competing interest

The authors declare that they have no competing interests.

## Authors’ contributions

MRM and MAM contributed to the design of the study, the analysis of the data and the interpretation of the results. MRM contributed to the development of the arrays and has conducted the experiments. JFA contributed to the analysis of the data and the interpretation of the results. All the authors have revised the manuscript and have given their final approval for publication.
